# Supplementary figures and images for: Bayesian model selection for multilevel models using integrated likelihoods
Source: PLoS One. 2023 Feb 15;18(2):e0280046. doi: 10.1371/journal.pone.0280046 (PMC9931113; doi:10.1371/journal.pone.0280046)

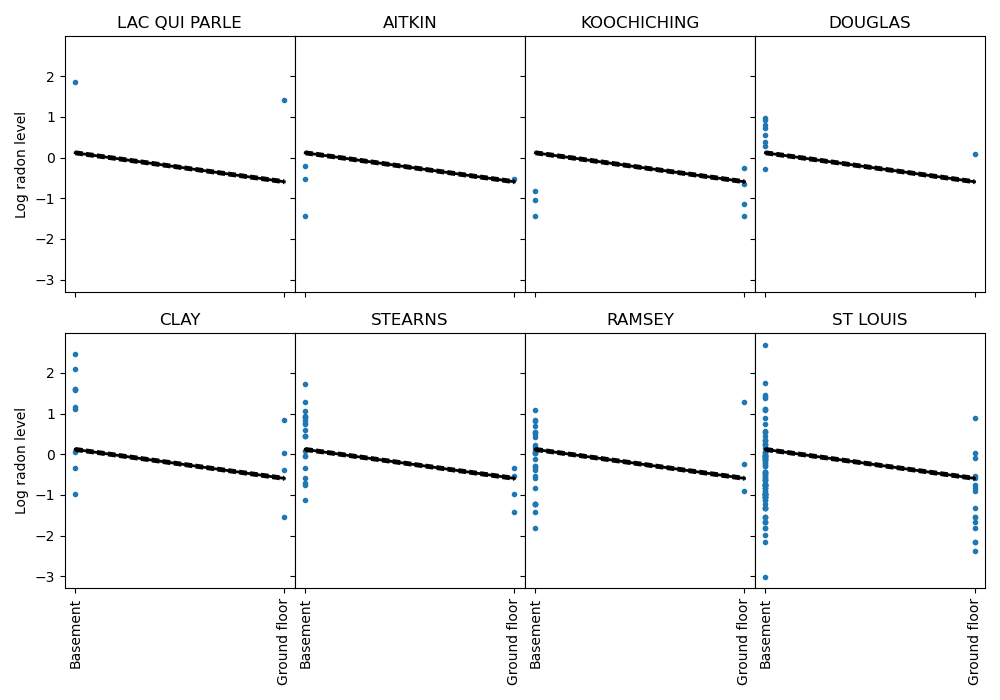

Supplement: S1 Fig — Each dot represents a measurement at either basement or ground floor level. The format of the figure follows [16], with the same counties represented, though we have standardised the log radon and uranium levels, so the y-axis scale is slightly different. The model fit is from the integrated likelihood sampling, and is shown as a gradient line from basement to ground floor, with one standard deviation from the mean in dotted lines. (TIF) [file pone.0280046.s001.tif]

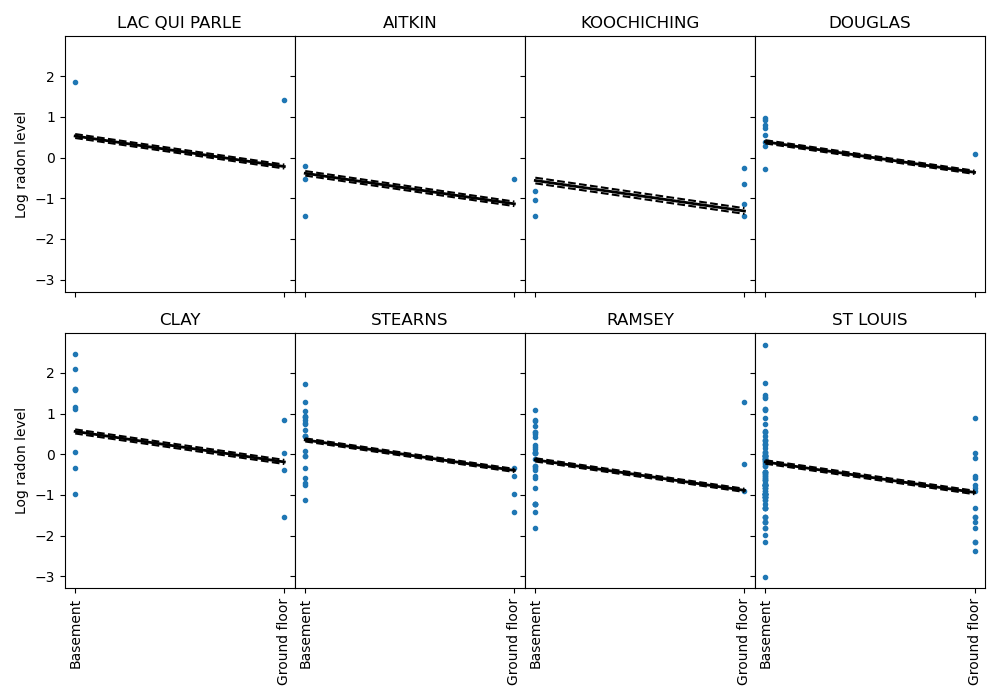

Supplement: S2 Fig — (TIF) [file pone.0280046.s002.tif]

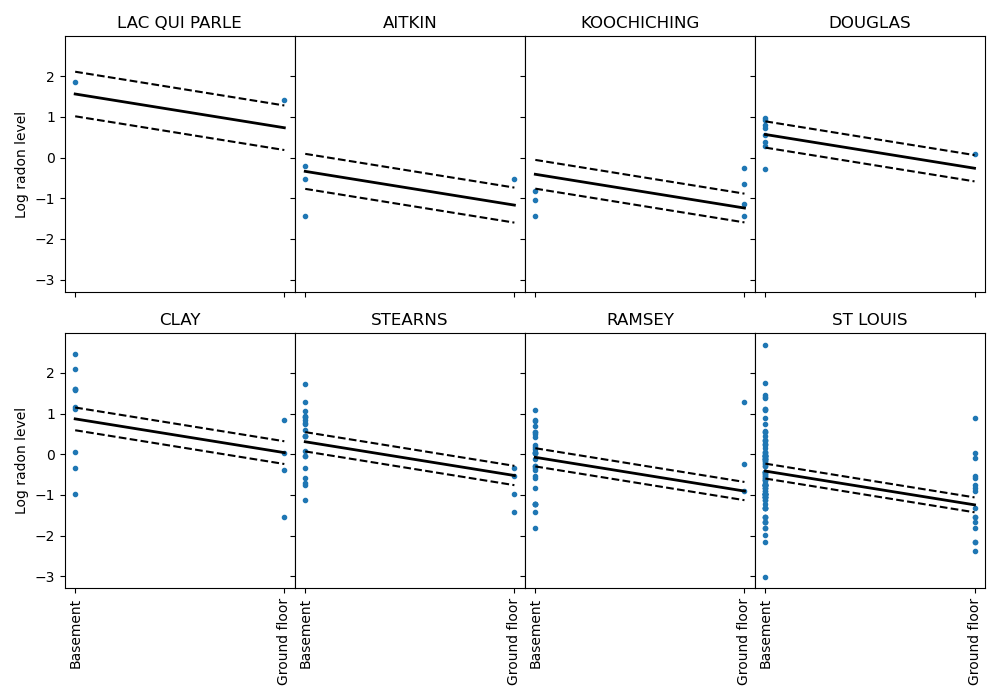

Supplement: S3 Fig — (TIF) [file pone.0280046.s003.tif]

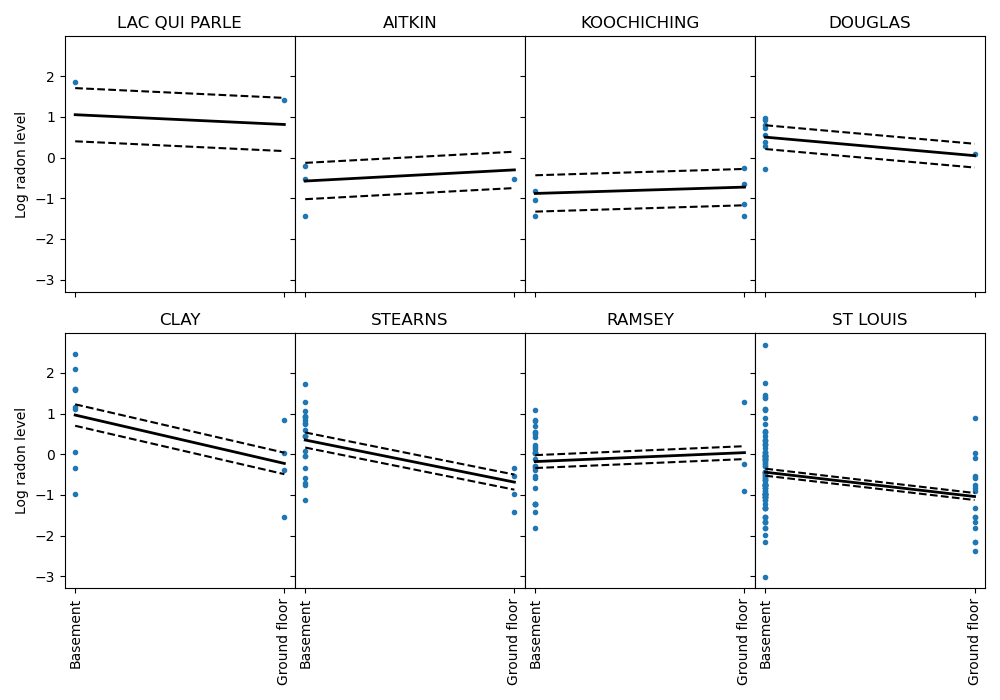

Supplement: S4 Fig — (TIF) [file pone.0280046.s004.tif]

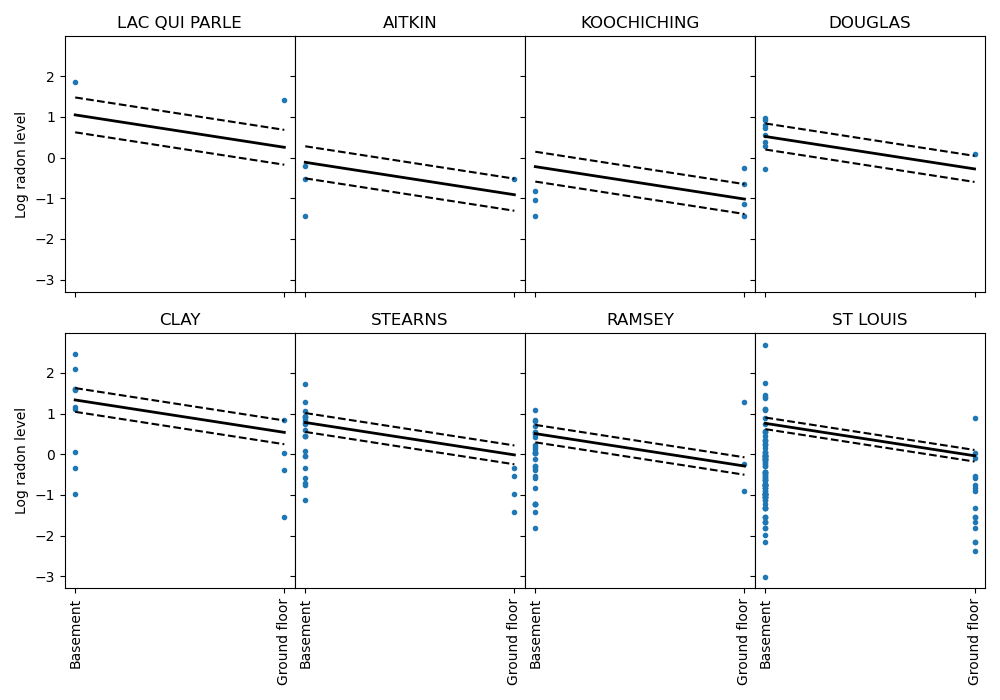

Supplement: S5 Fig — (TIF) [file pone.0280046.s005.tif]

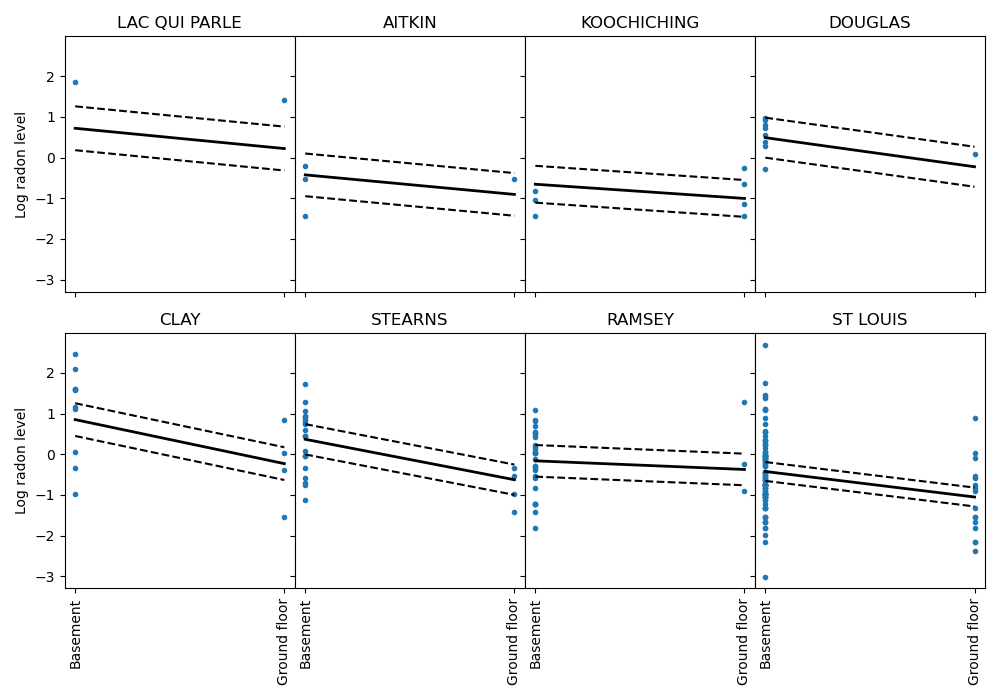

Supplement: S6 Fig — (TIF) [file pone.0280046.s006.tif]
