# Supplementary material for: Bayesian model selection for multilevel models using integrated likelihoods
Source: PLoS One. 2023 Feb 15;18(2):e0280046. doi: 10.1371/journal.pone.0280046 (PMC9931113; doi:10.1371/journal.pone.0280046)
Supplement: Attachment — Submitted filename: PLOS One response to reviewers-2.pdf [file pone.0280046.s007.pdf]

Reviewer #1: Review on “Bayesian model selection for multilevel models using marginal likelihoods”

This article proposes the use of marginal likelihoods instead of full likelihood to perform model selection for multilevel models. The methods are illustrated using generalized linear models where the responses are normally distributed, and the identity link is used. In this setting, the authors demonstrate that marginal likelihoods can be obtained analytically by integrating out the fixed and random effects. This reduces the dimensionality when computing the model evidence using SMC as only the variance parameters remain to be integrated out.

The manuscript is generally very well written with clear explanations although there are some typographical errors to be corrected. Below are some comments and suggestions for the authors’ consideration in revising the manuscript. A point-by-point response to these comments is appreciated.

1.I think using the term “marginal likelihood” to represent what is obtained after integrating a subset of the parameters is rather confusing because this term is usually taken to mean what is obtained after integrating out all the parameters. Perhaps “partial marginal likelihood” would be more accurate? Or the authors can consider other terms that provide a better description?

I’ve thought about this and I’m wary about getting the language right, as I believe ‘marginal partial likelihood’ and ‘partial marginal likelihoods’ are both different things in themselves. We decided to rename this to ‘integrated likelihood’, which has been used to denote this in previous works.

2.Page 5 line 151: Should ν be η in “integrated over β and ν”?

Corrected.

3.Page 5 line 170: Typo in “include the this using the notation above”

Corrected.

4.Page 5 line 172: Typo in “Rearranging the square brackets” because there are no square brackets in the expression above this line.

Corrected.

5.Page 6 line 176: Since the product is over i and j, I wonder if the power for $2 \pi \sigma_y$ should be nJ instead of just n? Same problem in equation (6).

No, this is correct as it is currently, though perhaps it could be made clearer, which I have done.

6.Page 6 line 177: A \sigma_\eta^2$ is missing in the numerator of the very last term.

Corrected.

7.Page 6 line 179: Typo in “rearranging for with β as in the”

Corrected.

8.Page 7 line 186: In line 4 of the equation, the power of $|\Sigma_\eta(\nu)|$ should be J instead of m. Similarly I wonder if the power of (2\pi\sigma_y) should be nJ instead of n? Same problem in equation (7).

Same as point 5, this is correct as it was.

9.There is only one example given on Minnesota radon contamination. Will the authors consider including a simulation study? For this real dataset, it is unclear how the models should be ranked since there is no true model.

This is a good suggestion, which we should have included from the start and have now implemented.

10.There is a lack of discussion on the comparison of SMC(marginal likelihood) and SMC(full likelihood). It is not clear what are the advantages of SMC(marginal likelihood)? Is the computation time reduced? How does this reduction depend on the number of observations and parameters? If this is the case, the authors should present some results on the timings? What other advantages are there? The authors need to present a clearer discussion of the advantages of their approach.

We did consider computation time but decided not to include it in the original manuscript. For the most part, it does offer a moderate improvement, but is of a similar order in magnitude. For the more complicated models where the derived likelihood is sufficiently complicated that sampling on the reduced parameter set takes longer than sampling all parameters. The advantage of this approach is primarily that we believe it is a better and more robust estimate of the model evidence than using all parameters, due to the difficulty in successfully sampling high-dimensional spaces. The simulation study confirms this.

11.Is it possible to design an experiment with a gold standard where the value of the likelihood can be computed? Then both approaches can be compared against this gold standard.

This is an interesting idea. I think the answer is no, if the true model is of the same form as those originally described. However, it is possible for a linear model with normal-inverse-gamma joint prior. To allow this comparison, we added this model in the simulation study, however we have also highlighted that the normal-inverse-gamma model is not generally a good model to choose. There are other approaches to computing the model evidence given the marginal likelihood we proposed, but no obvious rationale for choosing that over SMC.

Reviewer #2: This paper gives a derivation of the marginal likelihood for a general multilevel linear model using a specific prior setting. The derivations look accurate, and related literature is cited accordingly. I have the following comments:

1. Please fix "... the this ..." on line 170.

Corrected.

2. You can skip the equation before Eq. (5).

Not clear which part of the equation array you are referring to, but have removed the first line.

3. Lines 156 - 159, Σ_{η} is set to νI which implies that the elements of η are independent. What is the implication of this on the multilevel linear model? Is it a restrictive approach? Is it possible to do the derivations if the elements of η are not assumed to be independent?

This is an example of the form that the covariance could take, which we have not assumed in general. The point here is that since the covariance matrix is positive definite n_j \times n_j matrix, we can represent it as a vector of length < n_j * (n_j + 1) / 2, which is preferable for computational reasons. I have hopefully made this clearer.

4. Please link the result in Eq. (7) back to the elements given between lines 120 - 128. Or it would be good to provide an algorithm to outline the implementation of the derivation there in practice. This is only a part of the whole implementation and should be understandably by the practitioners.

The accompanying code is made open-access and available in order to allow implementation by other practitioners. I added a paragraph at the end of Methods to describe how the log marginal likelihood can be used in conjunction with any MCMC sampling scheme. I have also added an extra line after each showing the logarithm of the derived likelihoods, which is used in practice for computational reasons (e.g. underflow). I'm unclear what you are asking in terms of linking (7) back to lines 120-128, but I think the question is how to get from the (marginal) likelihood to the model evidence. This step is not part of our proposal, but I have added references relating to this.

5. The example lacks an informative data definition. Some descriptive statistics would help.

We have added some descriptive statistics.

6. Please give the data definition and the model's settings starting on line 203 in separate paragraphs for clarity. It is hard to follow in its current form.

Rewritten for greater clarity.

7. Once we get Table 1, what will we do with it? The link between the application and model selection is missing. What is the implication of your model selection effort? What does Minnesota radon contamination look like from the perspective of the best model selected by your approach?

We have added a figure showing the Minnesota radon contamination dataset using some of the models, following the same style as https://docs.pymc.io/en/v3/pymc-examples/examples/case_studies/multilevel_modeling.html.

8. What if I use an RJMCMC approach? Would I get different results in terms of the estimates of radon contamination? In that sense, what is the contribution of the work to the existing literature?

The idea of the manuscript is that by first marginalising out a subset of parameters, any subsequent method (particularly MCMC methods) used to estimate the model evidence will result in a more robust estimate, as the sampling is over a hugely reduced dimension. This includes SMC, RJMCMC, hierarchical MCMC i.e. Chib and Carlin. We are agnostic to the choice of method overlayed on top of this, choosing SMC for ease, but we anticipate similar results with other MCMC methods. We believe testing across a wider range of MCMC methods is beyond the scope of what this manuscript seeks to achieve, but the code can easily be adapted to any MCMC sampling method within the PyMC package (I don't believe this includes RJMCMC currently). The discussion has been amended to make this point clearer.

Thank you to both reviewers for your considered and detailed feedback.

Note to editor:

We have made some other minor changes, including updating and rerunning the code to use PyMC v4 instead of PyMC v3. This has resulted in some changes to Table 1 as, even when seeded, PyMC gives slightly different results when run multiple times.
